# Pro-MAP: a robust pipeline for the pre-processing of single channel protein microarray data

**DOI:** 10.1186/s12859-022-05095-x

**Published:** 2022-12-09

**Authors:** Metoboroghene Oluwaseyi Mowoe, Shaun Garnett, Katherine Lennard, Jade Talbot, Paul Townsend, Eduard Jonas, Jonathan Michael Blackburn

**Affiliations:** 1 Department of Integrated Biomedical Sciences, Division of Chemical and Systems Biology, Faculty of Health Sciences, University of Cape Town, Cape Town, South Africa; 2 Manchester Cancer Research Centre, Division of Cancer Science, Faculty of Biology, Medicine and Health, University of Manchester, Manchester, UK; 3 Faculty of Health and Medical Sciences, University of Surrey, Guildford, Surrey UK; 4 Surgical Gastroenterology Unit, Division of General Surgery, Groote Schuur Hospital, University of Cape Town, Cape Town, South Africa

**Keywords:** Protein, Microarray, Single channel, Protein microarray analysis, Pro-MAP

## Abstract

**Background:**

The central role of proteins in diseases has made them increasingly attractive as therapeutic targets and indicators of cellular processes. Protein microarrays are emerging as an important means of characterising protein activity. Their accurate downstream analysis to produce biologically significant conclusions is largely dependent on proper pre-processing of extracted signal intensities. However, existing computational tools are not specifically tailored to the nature of these data and lack unanimity.

**Results:**

Here, we present the single-channel Protein Microarray Analysis Pipeline, a tailored computational tool for analysis of single-channel protein microarrays enabling biomarker identification, implemented in R, and as an interactive web application. We compared four existing background correction and normalization methods as well as three array filtering techniques, applied to four real datasets with two microarray designs, extracted using two software programs. The normexp, cyclic loess, and array weighting methods were most effective for background correction, normalization, and filtering respectively.

**Conclusions:**

Thus, here we provided a versatile and effective pre-processing and differential analysis workflow for single-channel protein microarray data in form of an R script and web application (https://metaomics.uct.ac.za/shinyapps/Pro-MAP/.) for those not well versed in the R programming language.

**Supplementary Information:**

The online version contains supplementary material available at 10.1186/s12859-022-05095-x.

## Background

Protein microarray technology enables the simultaneous monitoring of expression levels for hundreds to thousands of proteins, to quantify their interactions and associated functions [1]. In particular, functional protein microarrays involve the immobilization of full-length functional protein targets or domains on a slide, which are incubated with a biological sample containing interacting molecules, such as autoantibodies [2]. They are increasingly being used in biomarker detection and drug discovery for various diseases, including cancers, where early detection is key to improved prognosis [3–5]. However, the translation of these microarray data into biologically significant conclusions requires automated data handling and processing, making it a crucial step. Tantamount to this, is the pre-processing and normalization of the extracted signal intensities from the microarrays. Several strategies have been proposed to overcome the analytic difficulties derived from fluorescence overlapping and background noise; however, no real consensus has been reached [6–9].

The design of high-throughput protein microarrays is based on, and therefore bears several similarities to, the established gene expression microarrays. As a result, researchers have often adapted methodologies and computational tools originally developed for gene expression microarrays to protein microarrays. However, as has become increasingly apparent, there are several distinct features that distinguish both arrays and should subsequently affect their analysis. For instance, most protein arrays have much fewer probes than gene expression arrays [10, 11] thus, more caution may be taken when determining if probes in the former should be discarded. Furthermore, inter-individual differences in protein activity are ubiquitous; thus, the dimensionality problem and the distinct biological nature of the data may make computational tools made for gene expression arrays unsuitable for protein arrays [12].

To the best of our knowledge, there are currently three major tools available for single channel protein microarray analysis: Protein Array Analyzer (PAA) [13], Protein Microarray Analyzer (PMA) [14], and a Protein chip analysis tool (ProCAT) [15]. However, there are conflicting themes between these analyzers in terms of methods used for pre-processing the microarrays; this precludes comparisons and data collaborations. In terms of pre-processing analyses, the PAA is a flexible pipeline that conducts *normexp* background correction and batch filtering on provided data. It also offers the user with option of comparing MA plots to determine which normalization techniques (cyclic loess, vsn, or quantile) best suit the data. However, this pipeline requires intermediate programming skills at least, thereby limiting the user pool. In contrast, PMA is built in JAVA and can be used via a fairly simple graphical user interface. It conducts neighbourhood background correction and net intensity correction, enables the user to define noise threshold and replicate CV thresholds, and finally runs intensity and quantile normalization amongst sub- and whole arrays. Finally, ProCAT uses neighbourhood background correction, data filtering, and scale normalization to pre-process microarray data. However, with these last two pipelines, there is no justification provided as to why these methods have been selected above other available methods. Furthermore, these tools have several limitations, including the file formats recognized and array layouts accepted by the pipelines, which we address in our current pipeline.

In this study, we explore the various methods available for the analysis of single channel protein microarray data, largely focusing on background correction, normalization, and array filtering. Based on our findings, we have developed a robust and flexible statistical pipeline suitable for use in the pre-processing of all single channel protein microarrays regardless of array design, data extraction software used, or file format. The proposed single-channel Protein Microarray Analysis Pipeline (Pro-MAP) follows the logical steps involved in the analysis of microarray data [6, 16] and as such, outlines the following processing steps: (a) Data extraction, (b) Spot filtering, (c) Background correction, (d) Normalization, (e) Array filtering, and (f) Data consolidation. Steps (a) to (e) were conducted using the limma package in R [17]. To the best of our knowledge, this is the first study that objectively tests several popular methods used in each of the critical steps to select the most suitable ones for our protein microarray pre-processing pipeline.

## Results

To develop our pipeline, we compared four datasets, with two array designs, extracted using two image software programs, producing two file types. We compared four background correction, four normalization, and three array filtering methods to determine the most effective combination for the pre-processing of single-channel microarray data.

### Comparison of background correction methods

**Fig. 1:**
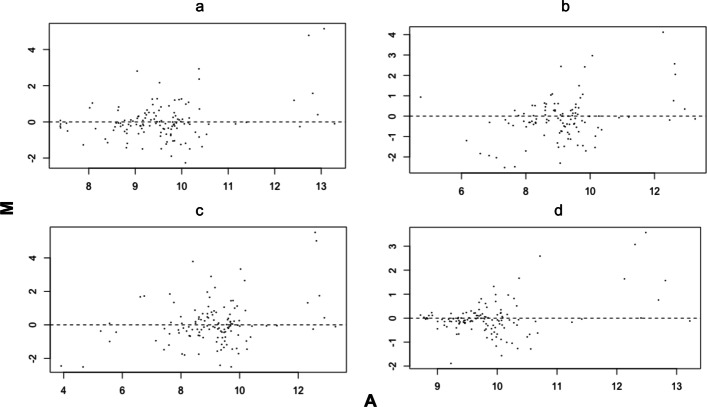
MA-plots obtained using different background correction methods on TR cohort arrays: **a** Rawdata, **b** Subtraction, **c** Movingminimum, **d** Normexp. A—average log intensity, M—expression intensity of array 1 versus the average of all the other samples

Following extraction of pixelated data from raw image files, the usual first step succeeding spot filtering in protein microarray data analysis involves the subtraction of the background signal from the foreground signals to generate net intensities for each spot. However, heterogeneity in the surrounding background signal makes background subtraction less robust than it might at first sight appear. Using MA (mean difference) plots, which depict differential expression, we therefore compared four different background correction methods on the PDAC technical replicates (TR) cohort dataset. The normalized M-values for one, randomly selected, array from the TR cohort, are shown in Fig. 1 and Additional file 1: Figures S1–S7. The arrays compared here represent patients with the same disease (pancreatic ductal adenocarcinoma; PDAC), sampled at two time points, so any differential expression should reflect inter-individual differences in autoantibody repertoires, as well as possible PDAC disease sub-groups, rather than being driven by the disease state per se. Thus, a background correction method resulting in a high offset and low M-value variability is preferable because the true M-values should be close to zero.

The differences between the background correction methods for the same raw data are conspicuous. Interestingly, some background correction methods produce more variable M-values than others, particularly at low A values. The M-values for the arrays were as large as 6.21, 6.06, 5.94, and 5.625 for rawdata, movingminimum, subtraction, and normexp, respectively (Fig. 1a–d). Furthermore, the hidden cost of standard subtraction and, to a lesser extent, the movingminimum method, which is not portrayed in Fig. 1, is the missing values. Across all 8 arrays of the TR cohort, 15.6% and 1.28% of the M-values were missing for the subtraction and movingminimum methods, respectively. The other methods gave no missing values.

Based on these data we were able to place the background correction methods on a continuum. At one end were methods that barely changed the foreground intensities, resulting in intensities offset from zero and relatively low M variability. On the other end of the spectrum, were methods, which changed the foreground intensities the most, resulting in a very wide range of intensities and a high M variability. The background methods could therefore be ordered by decreasing M variability: subtraction > movingminimum > rawdata > normexp.

### Precision of background correction methods

**Fig. 2:**
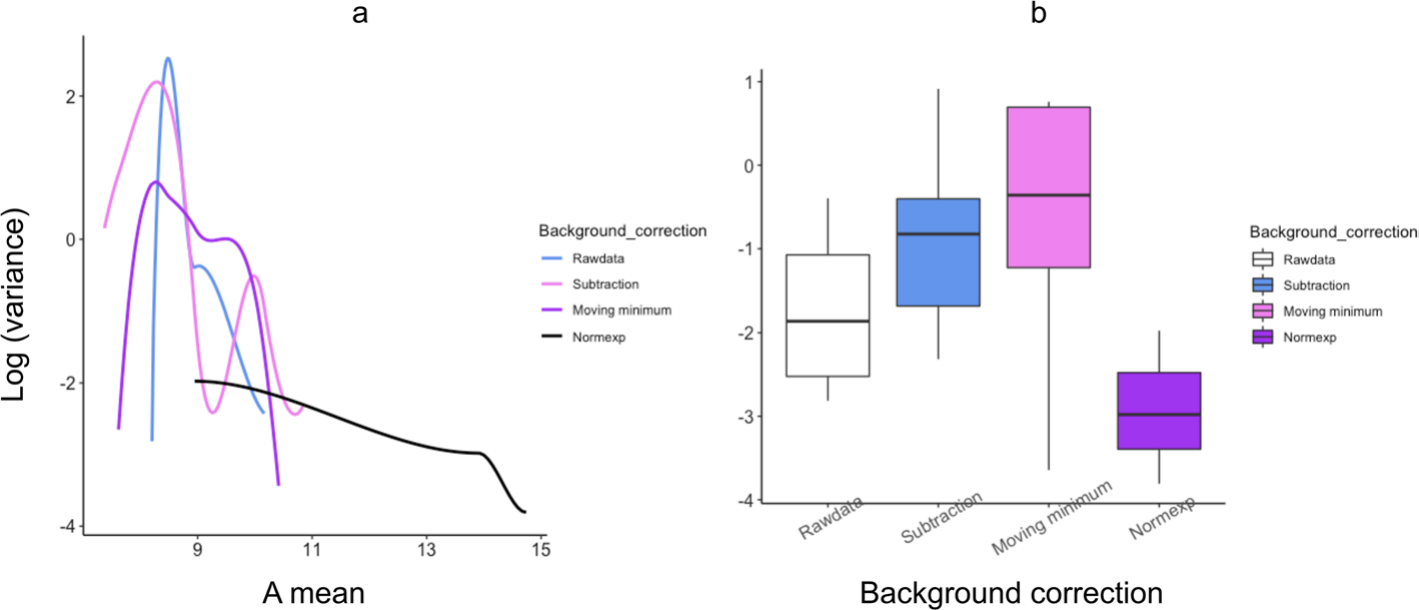
Residual variances from non-linear fits versus intensity for probes of JHB cohort arrays. **a** Line graph of smoothed log variances and **b** Boxplot showing range of log variances for each background correction method

The ‘JHB’ cohort, including PDAC and non-PDAC pancreatic cancer (PC) patient samples, was used to determine the precision of each background correction method relative to the dataset. The residual variance for each probe shows how accurately each expression value fits the proposed model. Figure 2 shows the trend in variability for each background correction method. For ease of comparability, the A values were standardized to be the same for each method. The vertical scale is log_2_-variance meaning that each unit on the vertical axis corresponds to a 2-fold change in variance. A lower variance suggested a better fit to the model and thus, increased precision. Hence, the correction method with the highest precision was identified as superior.

Most of the background methods showed low variance denoting a high precision, with normexp background correction showing the highest precision. No background correction resulted in the highest variance and lowest precision (Fig. 2a, b). The background correction methods could therefore be ordered from lowest to highest precision as rawdata < subtraction < movingminimum < normexp. Thus, based on both M variability and precision considerations, normexp was the background correction method chosen for our pipeline.

### Comparison of normalization methods

As a prelude to data analysis, it is common to normalize protein microarray datasets, in order to ensure that subsequent differential expression is focused on true biological signal and not skewed by systematic technical variation in signal intensities. However, the differing underlying structure of protein microarray datasets means that normalisation methods which are commonly used in genomic experiments are not necessarily well suited to normalisation of protein microarray datasets. We therefore compared the performance of four different normalization methods in order to objectively identify the most appropriate method for inclusion in our pipeline.

### Variability within replicates of normalized data

**Fig. 3:**
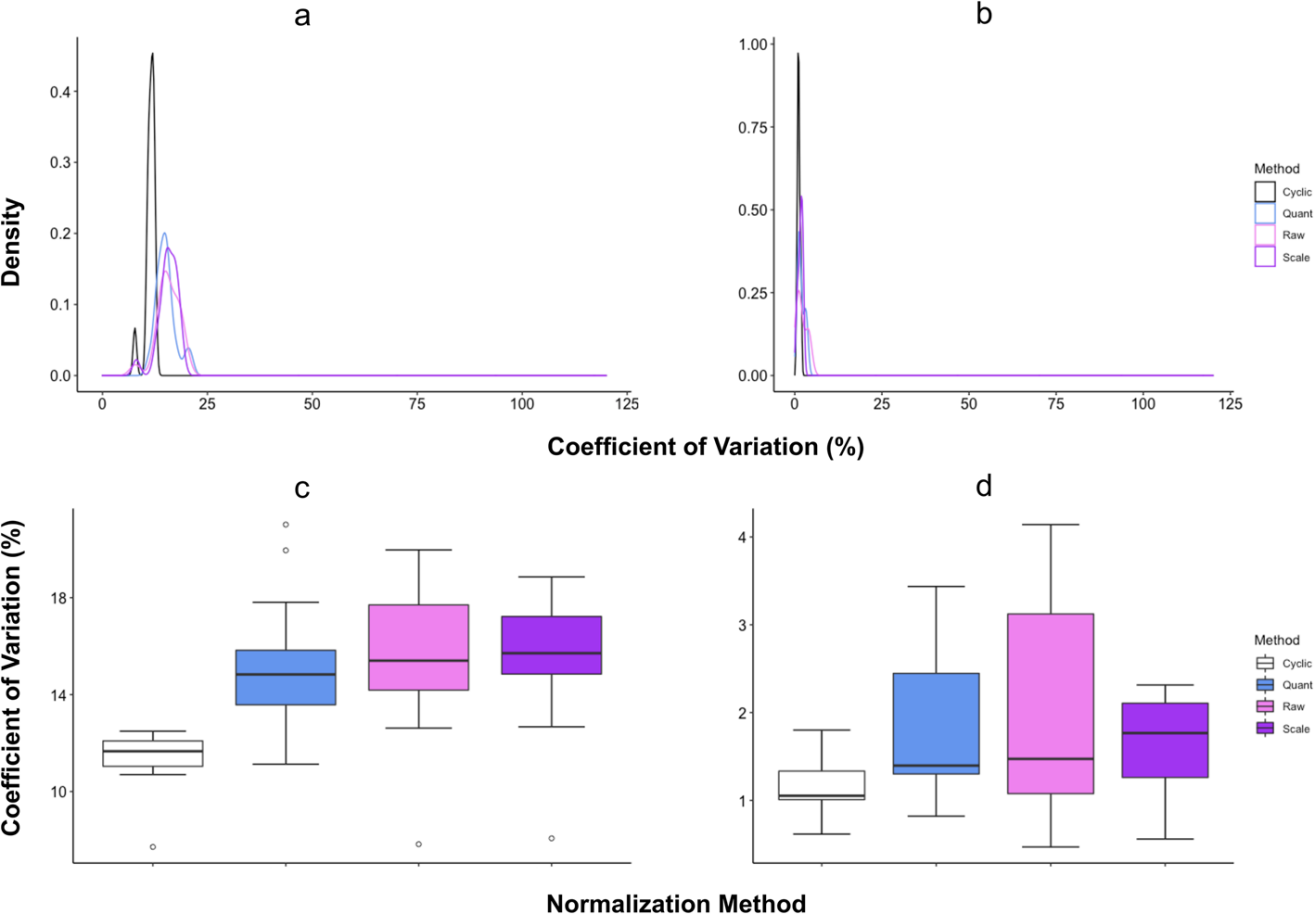
Comparison of the variability of the normalized data in the three technical replicates of all AlexaFluor-BSA controls from JHB cohort and the first three IgG controls from the GSH cohort. Density plot of coefficient of variation for selected control triplicates across all four normalization methods in the **a** JHB and **b** GSH cohorts. Boxplot of coefficient of variation across normalization methods in **c** JHB and, **d** GSH cohorts

The datasets compared here were background corrected using the normexp method available in limma, based on the findings above. We calculated the coefficient of variation (CV) of the normalized intensity values for all the AlexaFluor-BSA controls for each of the arrays in the JHB cohort and the first three IgG controls in the GSH (PDAC versus chronic pancreatitis [CP]) cohort. A low CV between controls of known concentrations denotes minimal systematic bias and technical variation, suggesting a better performing normalization method. We therefore compared the CVs of the controls after application of no normalization, scale, quantile, and cyclic loess normalization methods. Cyclicloess normalization resulted in replicate data with the lowest CVs (Fig. 3c, d) suggesting this method performed better, in terms of minimizing technical variability, than the other normalization methods evident by its more left-leaning peak for both cohorts (Fig. 3a, b).

Subsequently, we ran a one-way ANOVA comparing the means of the CVs for each normalization method in both cohorts (Additional file 1: Table S3). We found that there was a significant difference between methods (*p* < 0.001), for the JHB cohort alone. To further investigate these differences, we ran a Tukey’s multiple pairwise comparisons test and found that the significant differences in CVs were between cylicloess normalization and the other three normalization methods (*p* < 0.001). Thus, based on these data, cyclic loess normalization was the method selected for our Pro-MAP pipeline.

### Comparison of array filtering methods

In an ideal protein microarray experiment, all microarrays should be of equal quality, with uniformly low and homogeneous background, enabling true signal to be quantified. In reality, assay and washing artefacts can result in variable array quality, which, if not recognised, can bias downstream data analysis through artificial outlier effects. Visual inspection of individual raw array images can be used to identify low quality arrays that can be flagged or discarded prior to data analysis. However, this approach lacks objectivity. Here, we therefore used array weights to objectively quantify array quality and then compared three methods to address array quality in subsequent data analyses.

**Fig. 4:**
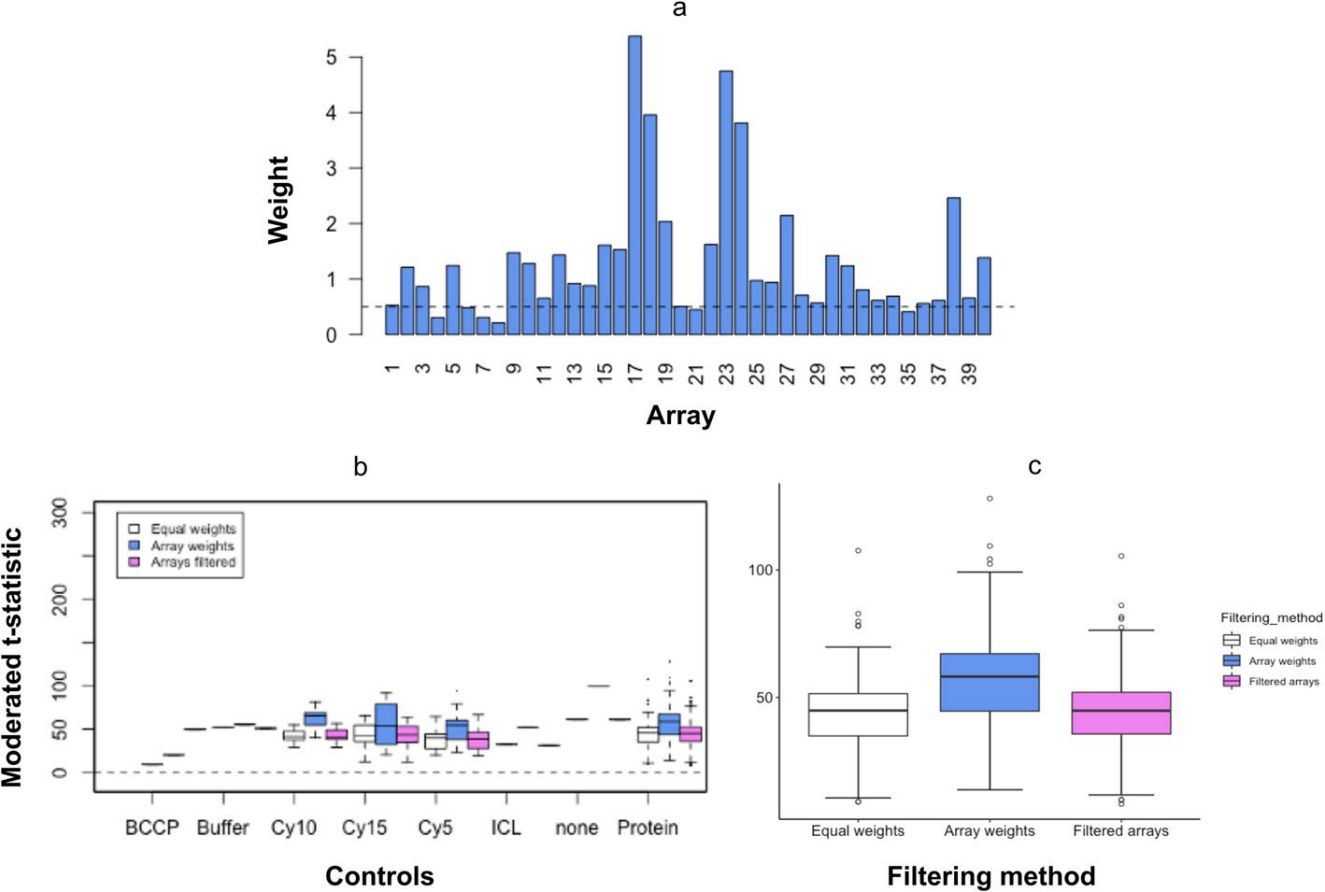
Comparison of different array filtering methods on differential expression of data. **a** Array weights for the EUR cohort datasets. The dashed lines show the weights at 0.5. **b** moderated t-statistics for each control in the EUR cohort dataset where equal weights, array weight, or array filtering had been applied. **c** Boxplot plot comparing moderated t-statistics for dataset where, no weighting, array weight, array filtering has been applied

The EUR cohorts, which consisted of prostate cancer and benign prostate disease patients was used to compare array filtering methods. Array weights were calculated using the method of Ritchie et al. [18] where a heteroscedastic model was fitted to the expression values for each protein. This dispersion model was then fit to the squared residuals from the mean fit and set to have array specific coefficients, which were subsequently updated in REML scoring iterations. The final estimates were then converted to weights (Fig. 4). We found a few arrays with a very low weight that could be considered “bad” arrays to be discarded or down-weighted (Fig. 4a).

Prior to analysis, data was normexp background corrected and cyclic loess normalized. We calculated the moderated t-statistics for the controls of a dataset with filtered arrays where the two lowest weighted arrays were removed, equally weighted arrays where no array weights were considered, or weighted arrays using the array weights previously calculated (Fig. 4a). Higher moderated t-statistics portray an increase in statistical power to detect true differential expression and thus, a more effective filtering method. We found that incorporating array weights into differential expression analysis produced higher moderated t-statistics than the other two filtering methods (Fig. 4b, c). This suggested this method was best for detecting true differential expression between disease conditions.

**Fig. 5:**
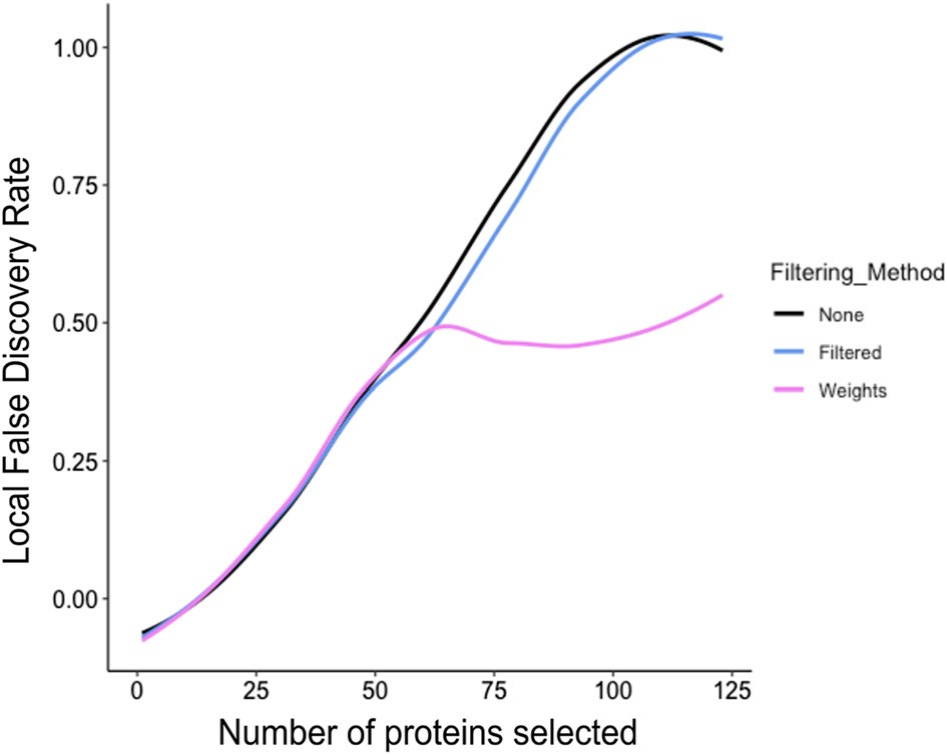
Local false discovery rate of dataset where equal weights, array weight, or array filtering have been applied. Local false discovery rate was calculated from *p* values adjusted using the Benjamin-Hochberg (BH) method

We then calculated the false discovery rates [19] of the dataset based on adjusted *p* values calculated using the Benjamini Hochberg Procedure. As expected, we found a lower false discovery rate, from arrays with 50 proteins and above, when array weights were incorporated in differential analyses whereas higher false discovery rates were shown when no array weights were used, or the two lowest weighted arrays were removed (Fig. 5).

### Final Pro-MAP pipeline

**Fig. 6:**
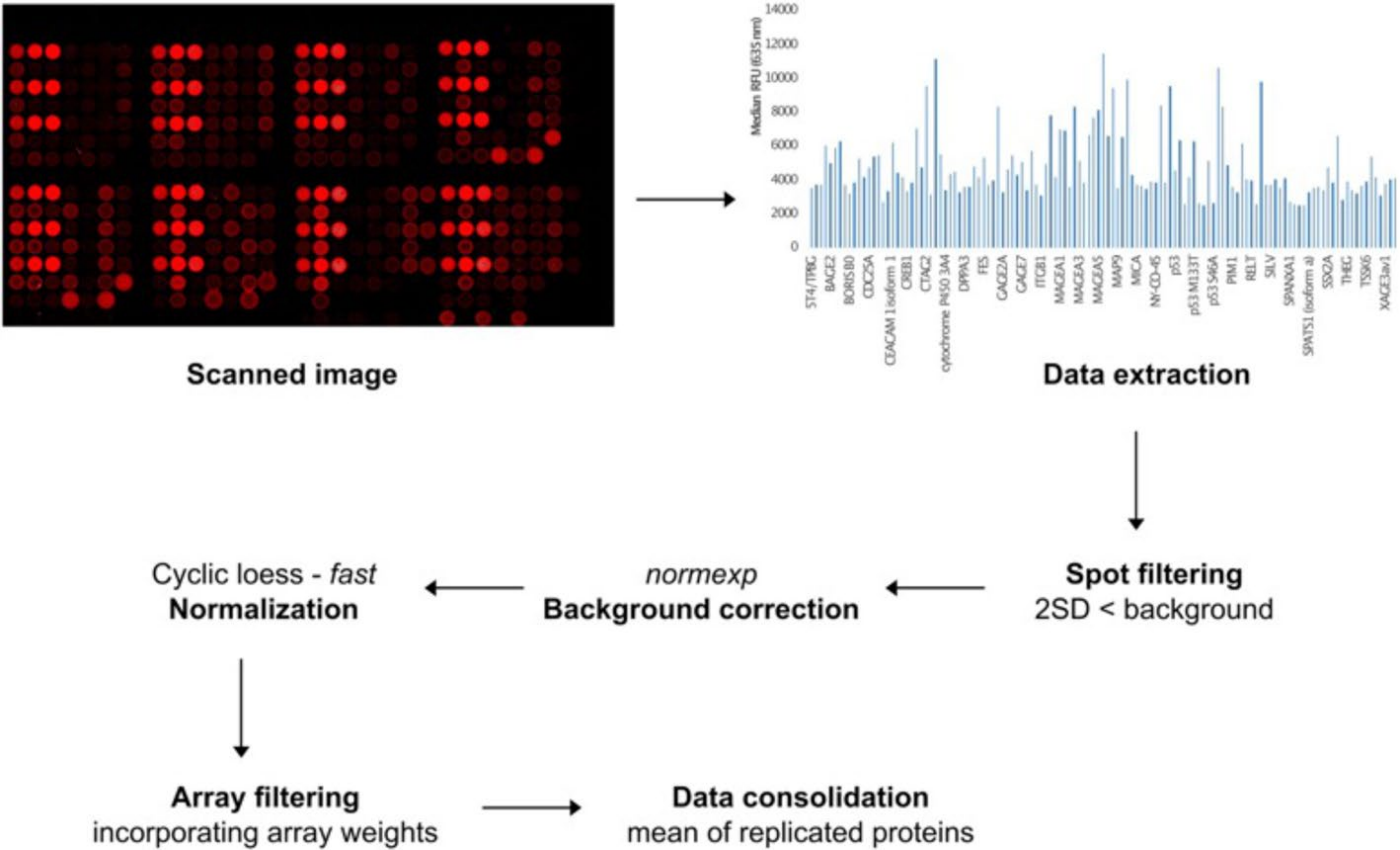
Schematic of proposed protein microarray analysis pipeline (Pro-MAP). The pipeline included data extraction, spot filtering, background correction, normalization, array filtering and data consolidation

Our final Pro-MAP pipeline begins with reading of data extracted from raw image files into R and spot filtering by discarding spots < 2SD of background. Subsequently, data is normexp background corrected and cyclic loess normalized. The array weights are then calculated to be used for downstream analysis and the means of replicate protein intensities on each array are calculated to condense the rows; all empty rows, and negative and positive controls are removed. The data are therefore consolidated into a dataset with columns representing each array or patient sample and rows representing probes or proteins (Fig. 6). From here, the researcher is free to analyse the data however they so choose.

### Implementation of Pro-MAP interactive web tool

**Fig.7:**
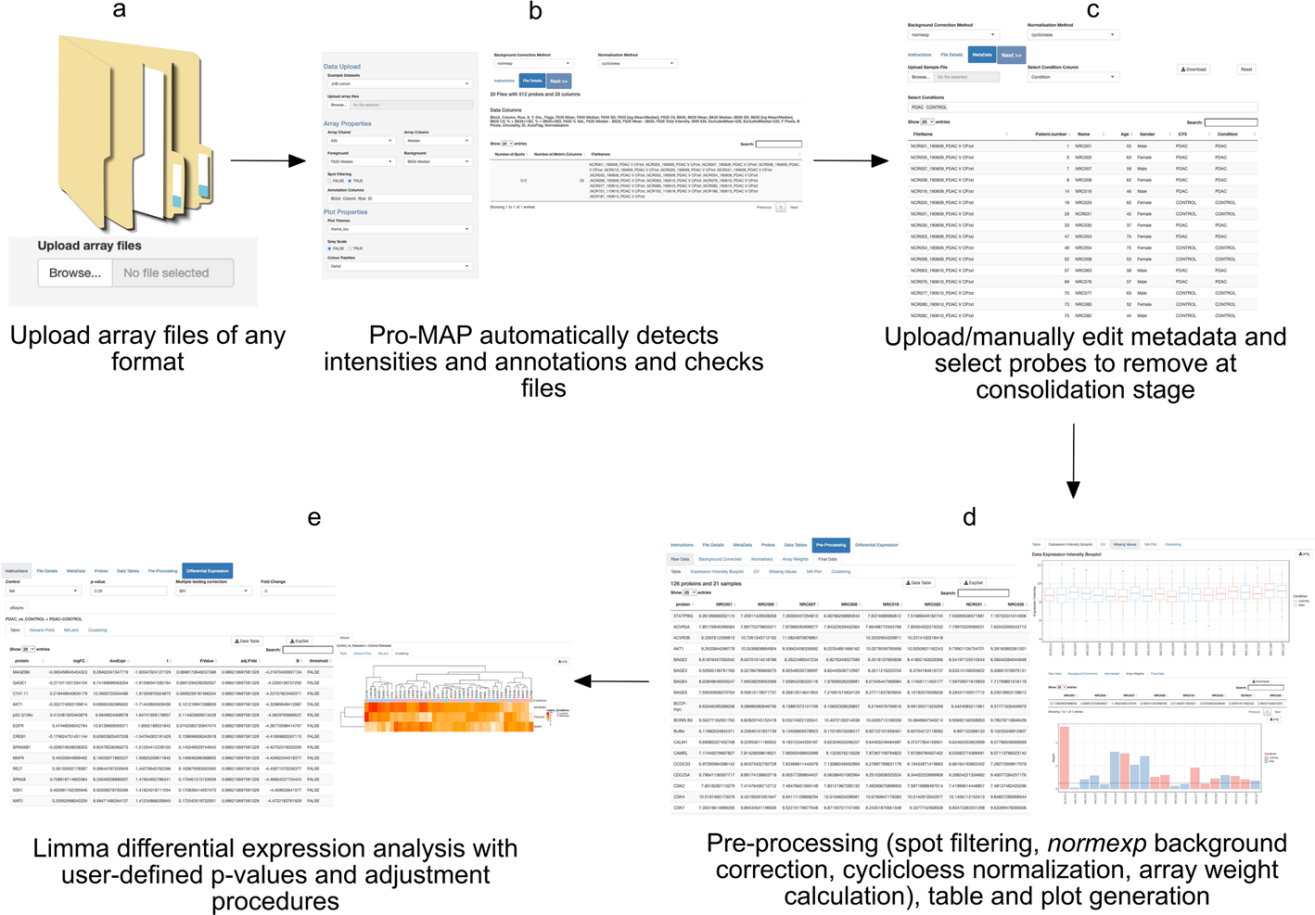
Pro-MAP pipeline. **a** Raw array files (of any type) are uploaded to Pro-MAP, **b** the shiny automatically detects foreground and background intensities and several annotations all of which can be manually edited by the user, **c** Metadata can be uploaded to the shiny for differential expression and data consolidation, control and/or empty spots to be removed at the consolidation stage can be picked from a drop down menu or an annotated spots file can be uploaded and spots to be removed can be chosen based on annotations, **d** data is background corrected and normalized, array weights are calculated, and data is consolidated and downloadable; plots are generated, **e** a limma differential expression analysis, using array weights, is run to identify potential biomarkers and these results are available as an expression set or txt file; a hierarchical cluster of differentially expressed proteins is generated and downloadable for the user. The figure was created using Affinity designer

From our pipeline, we built a complementary interactive web application which follows all the logical steps involved in the analysis of microarray data [6, 16] and as such outlines the following processing steps: (a) Data extraction, (b) Spot filtering, (c) Background correction, (d) Normalization, (e) Array filtering, (f) Data consolidation, and (g.) Differential expression analysis (Fig. 7).

### Data preparation

To start using Pro-MAP, fluorescent array signal readings that are output from any image analysis software (e.g., GenePix, Mapix, ArrayPro, etc.) as GPR or TXT files are uploaded into the Shiny app by either dragging and dropping files into the upload area or selecting them from the file system. Upon upload, default median foreground, background and annotation columns are automatically identified by the application but can be manually changed by the user based on preference. The main panel of the app is organized as tabs through which the user navigates to modify imputed data and view results. Subsequently, file details are automatically checked, and an error message arises if any disparate files are identified. The next tab produces a target file (metadata) with pre-set headers that can be downloaded and modified by the user to include disease conditions and any other defining factors deemed vital by the user for pre-processing and downstream analysis, then re-uploaded to match file names to column names in the final matrix. Metadata is also modifiable in the shiny. Following this, the user has the option of selecting probes to be removed at the final data condensation stage, this may include controls and empty slots. Alternatively, an annotated protein/probe list can be uploaded and probes to be removed selected based on a defined category e.g., “controls” (Fig. 7a, b).

### Pre-processing steps

Once the data has been prepared, spot filtering, background correction, normalization steps, and array weight calculations are performed automatically as described below, and plots are generated (Fig. 7c, d).

#### Spot filtering

On most arrays, non-empty spots are represented in triplicate or more to ensure the reliability of the data attained. A quality index for each spot is then used as a noise threshold below which undesirable spots, categorized as non-specific binding, are discarded. The noise threshold for Pro-MAP is determined as intensities < 2SD of the median background intensity and spots classified as such are discarded as “noisy”, represented as an ‘NA’ in the data table produced [14, 17].

#### Background correction

To further minimize the consequences of spatial heterogeneity across arrays due to processing effects, such as wash solution deposits or optical noise from the scanner, the data is normexp background corrected. This method uses a model-based adjustment based on a normal plus exponential convolution model fitted to the background subtracted signal. A maximum-likelihood estimation is then used to determine the corrected foreground intensities; thus, all corrected intensities are positive.

#### Normalization

To combat systematic experimental bias and technical variation in factors, such as sample labelling efficiency, scanner readout efficiency, and microarray quality without losing biological signals of interest, microarrays are normalized. In Pro-MAP, the data is “fast” cyclic loess normalized. Each array is normalized with a reference array, which is an average of all arrays, by applying a correction factor which is obtained from a loess curve fit through MA plots of the arrays.

#### Array weight calculation

Each array is weighted using an REML algorithm and weights are thereafter incorporated into downstream differential expression analysis models.

#### Pre-processing output files and plots

Following pre-processing, interactive plots of the expression intensities of the raw data, log intensities of the normalized data, array weights, and mean log expression intensities of the condensed data are generated in a colour scheme that is user-defined. A txt file of the array weights to be used for downstream differential analysis is downloadable by the user. Furthermore, the final pe-processed array dataset, consolidated into mean intensities with columns and rows representing arrays and proteins, respectively, and unwanted probes removed is downloadable as a data table or expression set. Finally, the plots are downloadable as png files.

### Differential expression analysis

The final step in the Pro-MAP pipeline is the optional differential expression for the identification of potential protein biomarkers (Fig. 7e). A linear model, using calculated array weights, is fit to the normalized microarray data to fully model the systematic part of the data and determine variability between the groups using the limma package in R [17, 20]. To determine variability in the data based on comparisons of interest, contrasts matrices are pulled from the metadata. Subsequently, an empirical Bayes method is used to moderate the standard errors of the estimated log-fold changes. The probability level used to determine differential expression can be manually selected by the user with *p* < 0.05 set as the default.

### Tool development

We developed the Pro-MAP web tool that covers all the necessary steps in the pre-processing and analysis of single channel protein microarrays. Its core has been implemented using R (version 4.0) using shiny[21], limma [17] for reading the array files and metadata, spot filtering, background correction, normalization array weight calculations, and differential expression analysis, and dplyr [22] for data consolidation. For plot generation ggplot2, ComplexHeatmap, plotly, EnhancedVolcano, and Biobase were used [23, 24, 25, 26, 27].

## Discussion

This study highlights some of the pitfalls of previously used pre-processing techniques as they pertain to single-channel protein microarrays. Here, using real datasets, commonly used pre-processing methods are compared to determine which are the most accurate for use in pre-processing single-channel protein microarrays. From this, we created a robust single-channel protein microarray analysis pipeline, Pro-MAP, in R.

Our study showed that the various background correction methods tested differed in terms of how accurately and precisely they portrayed the data. We compared no background correction, subtraction, moving minimum, and normexp background correction methods. We found, using a small technical replicate (TR cohort) dataset that where the log intensity should have been minimal, using the normexp background correction most accurately depicted this in terms of log intensity ratios (M) as it resulted in the smallest M values. Subsequently, we measured the precision of each correction method using residual log variances and found that the normexp correction method produced the lowest variances and therefore was the most “precise”. We also found that next to rawdata, the commonly used subtraction method was markedly worse than the other background correction methods. This is similar to findings by Ritchie et al. [28] who also showed that using this method resulted in a higher number of false discoveries.

To determine the optimal normalization methods for our arrays, we corrected the background of our dataset using the normexp method in limma. Subsequently, we compared the no normalization, scale, quantile, and cyclic loess normalization methods. We found that scale normalization resulted in the highest CVs of all the normalization methods suggesting it had the least ability to correct for technical variation and systematic bias in our data. In contrast, cyclic loess showed an apparent improvement over all other methods in terms of reproducibility, with CVs < 15% in both datasets used.

Finally, we tested the effect of equal array weights, array filtering of low weighted arrays, and the incorporation of array weights into differential expression analysis [18] on the ability to detect true differential expression in datasets without increasing FDR. We found that the moderated statistics were higher for datasets where array weights had been incorporated than those where the other two array filtering methods had been applied. We also found that FDR of the dataset where array weights had been used in downstream analyses was much lower than those to which the other two array filtering methods had been applied. These findings suggest that the inclusion of array weights in analyses may increase the ability to detect true differential expression.

Our pipeline provides a robust pre-processing analysis workflow for the preparation of single-channel protein microarray data for a variety of array designs, scanned and extracted using different scanner types and image analysis software programs. We filter the extracted data based on a noise threshold of < 2SD of the background. Subsequently, the data is normexp background corrected and cyclic loess normalized, and array weights are calculated. Finally, the data is consolidated into a dataset consisting of columns representing samples and rows representing proteins, with all the controls filtered out in readiness for downstream analysis. The use of a single pipeline for all single-channel protein arrays will facilitate future data sharing and research collaborations, which is currently limited in the field.

## Conclusions

From this pipeline, we have developed Pro-MAP, a state-of-the-art single channel protein microarray analysis pipeline with a user-friendly web interface. The results at each step, pre-processing and differential analysis, are downloadable, including publication-ready plots in user-defined colour themes. To encourage further development of single-channel protein microarray analysis and enable users to run their analysis locally at their personal devices, considering confidential patient level data is often analysed, the base R script (Additional file 2: Data S1) and web application code are made openly available. Future studies will include the addition of batch normalization to enable comparisons of data collected over several time frames and an archival system for the storage of single-channel protein microarrays, which remains a largely needed resource for the research community.

## Methods

In this study, we used four test datasets, and sample collection was approved by the University of Cape Town Human Research Ethics Committee (HREC 559-2018) and the Committee of Health and Social Care of Guernsey Ethics (IJG/C5.4). Moreover, written informed consent was obtained from all individuals, from which patient samples were derived for the study. All arrays were assayed as previously described [10, 29].

### Study cohorts

The datasets selected here were the most appropriate ones from the pool available to us. The first dataset included 4 PDAC patient samples assayed in 2019 and again in 2021. The arrays were printed using the CT100+ design developed by Blackburn et al., [10]. On this array, 123 proteins two negative controls, and five positive controls, three of which are known concentrations of AlexaFluor 647-Biotin BSA (5, 10, and 15 ng/µL) are printed in triplicates, in a 4-plex manner on in-house, streptavidin coated Nexterion H-slides (Material code: 1070936, Schott, Germany). These arrays were scanned on an Inopsys scanner (AL 4500, Inopsys) and the data was extracted using Mapix v 9.0.0 (Inopsys, France). The resulting dataset was used to compare MA values produced by the four most popular background correction methods used for single-channel microarray analysis (Additional file 1:Table S1). These data were ideal for MA plot comparisons because they included patients with the same disease and replicated thus, background correction was expected to present minimal differential expression.

The second dataset was derived from a Johannesburg (JHB) pancreatic cancer cohort consisting of 10 pancreatic ductal adenocarcinoma (PDAC) samples and 10 controls, which included other non-PDAC pancreatic cancer patient samples. The arrays were printed, scanned, and data was extracted similarly to the TR cohort dataset. This dataset was used to compare variance/precision between the four background correction methods as well as the control CVs produced by each of the four normalization methods compared (Additional file 1: Table S1). This dataset was comparable to a normal biomarker dataset containing a disease and control cohort and was therefore suitable to determine precision of our background methods. It also contained several positive control probes that could be used to compare the normalization techniques.

The third dataset was derived from a Groote Schuur, Cape Town (GSH) cohort consisting of four arrays each with five PDAC patient pools per array and two arrays each with four chronic pancreatitis (CP) patient pools per array. The arrays used were pre-printed Sengenics Immunome slides and each array consisted of 1622 distinct proteins with 7 negative and 29 positive controls, including Ig and Cy3 BSA proteins. These arrays were also scanned on an Inopsys scanner, and the data was extracted using the Mapix software. We used this data to determine the effects of the different normalization methods on the CVs of a different set of controls from those used in the CT100+ arrays to confirm our results (Additional file 1: Table S1).

The final and largest dataset consisted of a European (EUR) cohort consisting of 20 prostate cancer patients and 20 controls (benign prostate disease patients). The CT100+ design was used to print the arrays that produced this dataset and scanned using the Inopsys scanner. These data were extracted using GenePix Pro v 6.1 software (Molecular Devices, LLC). As the largest dataset, this was most suitable for comparison of the moderated t-statistics and false discovery rates produced by the three array filtering techniques compared as a removal of arrays did not significantly affect the data (Additional file 1: Table S1).

### Background correction methods

Background correction is essential for removing the effects of non-specific binding or spatial heterogeneity across arrays due to processing effects, such as wash solution deposits or optical noise from the scanner [30]. Here, we compared four background correction methods, available in limma, for single channel arrays (Table 1), which use different processing methods to remove background signals. All methods were implemented using the backgroundCorrect function of the limma software.

**Table 1:** Summary of background correction methods compared

Method	Bg estimate	Adjustment
None (R)	None	None
Subtraction (S)	Local Median	Subtraction
Movingminimum (M)	Neighbour-Median	Subtraction
Normexp (N)	Local Median	Model

#### None

Here, a background = 0 was set. Thus, the median foreground alone was used with this option.

#### Subtraction

This is the standard method of background correction and involves the subtraction of local background estimates from foreground intensities to provide, in theory, an unbiased estimator of the true signal due to hybridization [28]. In this study, the median foreground and background estimates were used. Notably, this method produces negative intensities when the background intensity is larger than the foreground intensity, resulting in missing or highly variable log-ratios and higher false discovery rates [31, 32, 33, 34]. To combat this issue, several downstream approaches often must be taken: filtering of low intensity spots, development of methods that incorporate variance-intensity dependence into differential analysis, or transformation of corrected intensities to stabilize the highly variable intensities.

#### Movingminimum

In this method, the background estimate for each spot is replaced by the minimum/lowest value of the spot of interest and the surrounding eight neighbours (3 × 3 grid of spots) which is then subtracted from the foreground. This is done to avoid skewed background intensities due to artefacts or dust particles [15].

#### Normexp

This method uses a model-based adjustment based on a normal plus exponential convolution model [30, 35]. The convolution model is fitted to the background subtracted signal and a maximum-likelihood estimation is used [28]. Thus, the expected signal, given the observed foreground, becomes the corrected intensity, resulting in a smooth, monotonic transformation of the background subtracted intensities, such that all corrected intensities are positive [35]. The normexp + offset, which is a slight variation of the normexp method whereby a positive offset (k) is added to move the corrected intensities away from zero was also considered. This is a simple variance stabilizing technique, which may reduce the usual variation of log-ratios at low intensities. Using 3 previous cancer protein datasets, we determined k by fitting linear models through data processed with 0 ≥ k ≤ 50 and the offset with the largest df.prior, representing the best stabilized variance and greater power to detect differentially expressed proteins, was chosen as optimal. For two of the three datasets, k = 0 produced the highest df.prior values (Additional file 1: Table S3). Thus, for our pipeline we only compared normexp with the other background correction methods.

### Normalization methods

Normalization accounts for systematic experimental bias and technical variation in factors, such as sample labelling efficiency, scanner readout efficiency, and microarray quality while maintaining biological signals of interest [36]. Several methods of normalization have been developed over the years, including global, scale, print-tip, various loess, and quantile methods [16, 37, 38]. However, most of these methods have been developed for gene arrays and their associated assumptions, which are not analogous with those of protein arrays [16]. Thus, making use of such methodologies could mask biologically relevant and interesting data without increasing reproducibility. We therefore compared three existing normalization methods available in limma for single-channel arrays. Data was normexp background corrected prior to each normalization step.

#### None

Here, the data was normexp background corrected and no normalization step was applied.

#### Scale

This method is the standard method of normalization for microarrays and is performed on a linear scale as opposed to a log scale. Scaling transforms each array so that the median for each array is equal [39, 40]. However, several studies have shown that non-linear methods, independent of the baseline array chosen, perform better.

#### Quantile

This is the most popular normalization method used in microarray studies due to its low computational load. This method is based on the idea that quantile–quantile plots show an equal distribution of two data vectors if the plot produces a straight line. Thus, the method “forces” an equal distribution for the probe intensities for each array in a dataset [38]. However, there is the possibility that in using this method some biologically significant information is suppressed and therefore lost in downstream analyses.

#### Cyclic loess

Cyclic loess normalizes two arrays at a time by applying a correction factor which is obtained from a loess curve fit through MA plots of the arrays. In this study, we utilized the “fast cyclic loess” method for our pipeline. This is a less aggressive method than the quantile normalization method and is a non-linear loess method where, in the case of the “fast method” arrays are normalized to a reference array, which is the average of all arrays [41]. In recent years, it has become the favoured method for the normalization of single-channel protein arrays.

### Array filtering methods

Despite background correction and normalization, variations in data quality may remain, affecting downstream analyses [18, 42]. Problems can be detected at the probe and array level; however, problems at the array level are more critical as a single low-quality or “bad” array may have significant effects on the microarray data acquired. Most established studies exclude these “bad” arrays from the dataset, but this is risky as the arrays may still contain some useful information about protein expression, which is embedded in a higher degree of noise than in high-quality or “good” arrays. Thus, Ritchie et al. [18] introduced a graduated, quantitative approach in which poorer quality arrays are down-weighted to be included in analyses. We compared three methods of array filtering to determine which was most suitable for microarray data.

#### Equal weights/no filtering

Following background correction and normalization, all arrays are used equally in the analyses.

#### Array weights

Here, following background correction ad normalization, the array weights are estimated using the REML algorithm created by Ritchie et al. [18] and these are incorporated in subsequent analyses.

#### Array filtering using array weights

Here, following background correction and normalization, the two arrays with the lowest calculated array weights/quality scores are removed prior to further analyses. It is important to note here that the cut-off for array filtering is investigator and data dependent. For this study, two arrays with the lowest weights were filtered out.

### Experimental design and statistical rationale

To compare background correction methods, we spot-filtered extracted data from the TR and JHB cohorts and applied all four background correction methods. For the TR cohort, we used the corrected intensities to generate mean difference plots and calculate the log intensity ratios (M) = log2(X − Y)/log2 Y and log intensity averages (A) = 1/2 (log2 X + log2 Y). As these were replicates of patients with the same disease, we expected the most effective background correction methods to have the smallest M and A values, denoting minimal differential expression between replicates. Subsequently, in the JHB cohort, we calculated residual standard deviation value or variance, (σ) which is a goodness-of-fit measure that shows how well a set of data points fit the actual model. The smaller the variance, the more predictive or “precise” and useful the model. The JHB cohort was quantile normalized as a normalization method had yet to be chosen and the data were analysed by fitting a probe-wise linear model to all arrays to estimate variability. The A-values were standardized to be the same for each correction method to enable comparability. The correction method with the smallest MA values and highest precision was chosen for the pipeline.

To compare our four normalization methods, the JHB and GSH cohorts were normexp background corrected following which all normalization methods were applied to the data. To determine which method best minimized technical variation and systematic experimental bias in the data, we calculated the coefficients of variation (CVs) of the AF Cy5 biotin BSA controls and IgG controls of the JHB and the GSH arrays, respectively. A lower CV of controls denoted a greater ability to minimize variation due to systematic experimental bias and technical variation without masking biologically relevant and interesting data. Thus, the method that consistently produced the lowest control CVs was used in the pipeline.

To compare our three filtering methods, we applied the most effective background correction and normalization methods, based on our comparative analyses, and each array filtering method to the EUR cohort data. We then compared moderated t-statistics for each class of probes among each method. A higher moderated t-statistic suggested an increase in statistical power to detect true differential expression without increasing the false discovery rate (FDR). To validate these findings, we then compared false discovery rates [19] for each of the three datasets produced from the array filtering methods being applied based on adjusted *p* values calculated using the Benjamin Hochberg adjustment [43].

All statistical analyses and plots were conducted and created using R (v 4.0) and ggplot2 [23], respectively.

## Supplementary Information

**Additional file 1**. Supplementary figures and tables cited in the text.

**Additional file 2**. A visual user guide ( file, which accompanies the Shiny app and is available online) highlighting the Pro-MAP methodology’s critical steps.

## Data Availability

The R script used to create the Pro-MAP shiny is available in the supplementary. The Pro-MAP shiny and all data needed to evaluate the conclusions in this paper are freely available at this link: https://metaomics.uct.ac.za/shinyapps/Pro-MAP/.

## Abbreviations

CP: Chronic pancreatitis
CV: Coefficients of variation
EUR: European
FDR: False discovery rate
GSH: Groote Schuur
JHB: Johannesburg
PAA: Protein Array Analyzer
PC: Pancreatic cancer
PDAC: Pancreatic ductal adenocarcinoma
PMA: Protein Microarray Analyzer
ProCAT: Protein chip analysis tool
Pro-MAP: Protein Microarray Analysis Pipeline
TR: Technical replicates
