# Supplementary material for: Pro-MAP: a robust pipeline for the pre-processing of single channel protein microarray data
Source: BMC Bioinformatics. 2022 Dec 9;23:534. doi: 10.1186/s12859-022-05095-x (PMC9733281; doi:10.1186/s12859-022-05095-x)
Supplement: Supplementary file 1 — Additional file 1. Supplementary figures and tables cited in the text. [file 12859_2022_5095_MOESM1_ESM.docx]

BMC Bioinformatics

Supplementary Material

Pro-MAP: A Robust Pipeline for the Pre-processing of Single Channel Protein Microarray Data

Metoboroghene Oluwaseyi Mowoe^1^*, Shaun Garnett^1^, Katherine Lennard^1^, Jade Talbot^2^, Paul Townsend^3^, Eduard Jonas^4^, Jonathan Michael Blackburn^1*^

^1^Department of Integrated Biomedical Sciences and Molecular Medicine, Division of Chemical and Systems Biology, Faculty of Health Sciences, University of Cape Town, Cape Town, South Africa.

^2^Manchester Cancer Research Centre, Division of Cancer Science, Faculty of Biology, Medicine and Health, University of Manchester, Manchester.

^3^Faculty of Health and Medical Sciences, University of Surrey, Guildford, Surrey, United Kingdom.

^4^Surgical gastroenterology Unit, Division of General Surgery, Groote Schuur Hospital, University of Cape Town, Cape Town, South Africa.

*Metoboroghene O. Mowoe; Email: [m_mowoe@yahoo.co.uk](mailto:m_mowoe@yahoo.co.uk)

*Jonathan M. Blackburn; Email: jonathan.blackburn@uct.ac.za

Supplementary Text

Table S1. Description of datasets used to test the various methods for preprocessing microarray data

| Dataset | Sample composition | Array design | Data extraction tool | Methods compared |
| --- | --- | --- | --- | --- |
| Technical replicates cohort (TR) | 4 PDAC patients (2019),  4 PDAC patients (2021) | CT100+ | Mapix v9.0.0 | Background correction methods  (MA values) |
| Johannesburg pancreatic cancer cohort (JHB) | 10 PDAC, 10 PC | CT100+ | Mapix v9.0.0 | Normalization methods (precision/variance and control CVs) |
| Groote Schuur, Capetown cohort (GHS) | 4 PDAC pools (5 patients each), 2 CP pools (4 patients each) | Sengenics Immunome | Mapix v9.0.0 | Normalization methods |
| European cohort (EUR) | 20 Prostate cancer, 20 benign prostate disease patients | CT100+ | GenePix Pro v 6.1 | Array filtering techniques (moderated t-statistics and false discovery rates) |

*PDAC – Pancreatic ductal adenocarcinoma, PC – non PDAC pancreatic cancers

Extra data for Method development

Fig. S1. MA-plots obtained using different background correction methods on TR cohort arrays: a.) Raw data, b.) Subtraction, c.) Moving minimum, d.) *Normexp*. A – average log intensity, M – expression intensity of array 2 versus the average of all the other samples.

Fig. S2. MA-plots obtained using different background correction methods on TR cohort arrays: a.) Raw data, b.) Subtraction, c.) Moving minimum, d.) *Normexp*. A – average log intensity, M – expression intensity of array 3 versus the average of all the other samples

Fig. S3. MA-plots obtained using different background correction methods on TR cohort arrays: a.) Raw data, b.) Subtraction, c.) Moving minimum, d.) *Normexp*. A – average log intensity, M – expression intensity of array 4 versus the average of all the other samples

Fig. S4. MA-plots obtained using different background correction methods on TR cohort arrays: a.) Raw data, b.) Subtraction, c.) Moving minimum, d.) *Normexp*. A – average log intensity, M – expression intensity of array 5 versus the average of all the other samples

Fig. S5. MA-plots obtained using different background correction methods on TR cohort arrays: a.) Raw data, b.) Subtraction, c.) Moving minimum, d.) *Normexp*. A – average log intensity, M – expression intensity of array 6 versus the average of all the other samples

Fig. S6. MA-plots obtained using different background correction methods on TR cohort arrays: a.) Raw data, b.) Subtraction, c.) Moving minimum, d.) *Normexp*. A – average log intensity, M – expression intensity of array 7 versus the average of all the other samples

Fig. S7. MA-plots obtained using different background correction methods on TR cohort arrays: a.) Raw data, b.) Subtraction, c.) Moving minimum, d.) *Normexp*. A – average log intensity, M – expression intensity of array 8 versus the average of all the other samples

Table S2. df.prior values from 3 previous cancer datasets using *normexp* offsets ranging from 0 to 50

| **Dataset** | **k = 0** | **k = 10** | **k = 20** | **k = 30** | **k = 40** | **k = 50** |
| --- | --- | --- | --- | --- | --- | --- |
| **1** | 5.308 | 5.319* | 5.301 | 5.268 | 5.228 | 5.468 |
| **2** | 7.866* | 7.539 | 7.182 | 6.847 | 6.545 | 6.406 |
| **3** | 1.585* | 1.468 | 1.389 | 1.331 | 1.287 | 1.252 |

k = positive offset added to move corrected intensities away from zero

df.prior = stabilized variance, which is correlated to ability to detect differentially expressed protein

*highest df.prior value

Table S3.

Descriptive statistics comparing CVs for the different normalization methods

| Normalization Method | Mean | SD | Normalization Method | Mean | SD |
| --- | --- | --- | --- | --- | --- |
| JHB COHORT | | | **GSH COHORT** | | |
| Rawdata | 14.3 | 4.17 | Rawdata | 0.626 | 0.285 |
| Scale | 13.7 | 4.36 | Scale | 0.621 | 0..271 |
| Quantile | 13.1 | 4.14 | Quantile | 0.924 | 0.287 |
| Cyclic loess | 10.4 | 2.22 | Cyclic loess | 0.616 | 0.275 |

**Data S1.**

R script for Pro-MAP pre-processing analysis pipeline

library("limma")

library("tcltk")

library("ggplot2")

library("tidyverse")

**#---------------------------Read in data and filter spots----------------------------------------------------**

#first make a targets file (.txt) with the following columns

#Name, filename, and Disease state (include any other extra information if necessary)

targets <- readTargets((file.choose())) #Read in target file

#write in function for calculating spot weights for filtering

spotfil <- function(x){

threshold = 2*sd(abs(x[,"B635 Median"]))

okred <- abs(x[,"F635 Median"]) < threshold*abs(x[,"B635 Median"])

+ as.numeric(okred)

}

**#---------------------------------------Read in image data files-----------------------------------**

# E – column name for median intensity for the foreground spots in .txt or .gpr file

# Eb – column name for median intensity for the background spots .txt or .gpr file

E <- read.maimages(tk_choose.files(),

columns=list(E=" ", Eb=" "),

annotation=c("Block", "Column", "Row", "ID", "Name"), #user can modify annotations

wt.fun=spotfil)

**#-----------------------------------------------Spot filtering------------------------------------------**

E_filter <- E$E * E$weights

E_filter[E_filter == 0] <- NA

E$E <- E_filter

**#----------------------------Visualization of Raw data---------------------------------------------**

boxplot(data.frame(log2(E$E)),

main = "Rawdata",

ylab="Expression Intensity",

col = "white",

font =12,

frame = FALSE)

**#---------------------------Background correction----------------------------------**

E_corr <- backgroundCorrect(E, method = "normexp")

**#---------------------------------Normalization------------------------------------**

E_norm <- log2(normalizeCyclicLoess(E, method = "fast"))

rownames(E_norm) <- paste(E$genes$ID) #ID represents column name of protein IDs/names

colnames(E_norm) <- c(targets$Name) #Name represents column name of array identifiers

**#----------------------------Visualization of Normalized data---------------------------------------------**

boxplot(data.frame(log2(E$E_norm)),

main = "Rawdata",

ylab="Expression Intensity",

col = "white",

font =12,

frame = FALSE)

**#---------------------------------Array weights----------------------------------**

arrayw <- arrayWeights(E_norm)

barplot(arrayw, xlab="Array", ylab="Weight", col="cornflowerblue", las=2)

abline(h=0.5, lwd=1, lty=2)

**#---------------------Condense and clean up data--------------------------------**

data <- tibble::rownames_to_column(as.data.frame(E_norm), var = "Proteins")

data <- data %>% mutate_at(vars(2:ncol(data)), as.numeric)

data$Proteins <- gsub("\\.[[:digit:]]*$", "", data$Proteins)

data <- as.data.frame(data) %>% group_by(Proteins) %>%

summarise_all(funs(mean))

drops <- c("control 1", "control2", "control3" etc.)

#Name all your controls and empty spots to be dropped from the condensed dataset

data <- tibble::column_to_rownames(data, var = "Proteins")

data <- data[!(row.names(data) %in% drops), ]

colMeans(is.na(data)) #optional – check %missing values

**#----------------------------Visualization of Condensed dataset-----------------------------------------**

boxplot(data.frame(data),

main = "Normalized",

ylab="Expression Intensity",

col = "white",

font =12,

frame = FALSE)
