# Supplementary material for: Pro-MAP: a robust pipeline for the pre-processing of single channel protein microarray data
Source: BMC Bioinformatics. 2022 Dec 9;23:534. doi: 10.1186/s12859-022-05095-x (PMC9733281; doi:10.1186/s12859-022-05095-x)
Supplement: Supplementary file 2 — Additional file 2. A visual user guide ( file, which accompanies the Shiny app and is available online) highlighting the Pro-MAP methodology’s critical steps. [file 12859_2022_5095_MOESM2_ESM.pdf]

The **single channel Protein Microarray Analysis Pipeline (Pro-MAP)** enables the easy upload of antibody array data in various file formats. Users can view the raw data, perform spot filtering, background correction, normalization, data consolidation and differential expression analysis.

Side Panel

This section refers to the grey panel to the left, which contains file input settings and plot display properties.

Data Upload

Array files, in .txt or .gpr format, and of any wavelength, can be selected from a file system or dragged into the browser by users.

- **Example Datasets**: Four example datasets, showcased in the linked paper (Mowoe et al., 2022), are available here to explore app functionality.

Array Properties

Array information, such as foreground, background and ID columns, is extracted from uploaded data and is available in a dropdown menu. The default columns used are set to median foreground and background and are editable by the user.

- **Array Chanel**: This refers to the fluorophore wavelengths used to visualise the probes.
- **Array Column**: The measurement column used for foreground and background (default is median), determined by the image extraction software used.
- **Foreground** and **Background** : Expression intensity columns used for microarray analysis
- **Spot Filtering**: If set to 'TRUE", this removes all probes that have a foreground intensity that is less than 2 standard deviations of the associated background.
- **Annotation Columns** : Additional identifier columns for each array e.g., ID, Flags, Uniprot ID etc.

Plot Properties

Colour palettes and/or themes for plots generated in the app.

- **Plot Themes**: a selection of ggplot themes that can be used
- **Grey Scale**: Creates greyscale plots regardless of themes chosen (default is set to FALSE)
- **Colour Palettes** : a selection of RColourBrewer pallets.

MainPanel

The Main Panel to the right of the aforementioned Side Panel, displays the array processing outputs. The Main Panel is arranged as a series of tabs, which appear sequentially as sections of the analysis are completed. Click on the **Next >>** tab to navigate through processing steps.

File Details

Following the upload of data to the app, array files are checked for consistency. Information on the number of array files uploaded, number of probes, as well as annotation columns is displayed. If there are any errors or inconsistencies in the uploaded file, warnings (orange) or errors (red) are shown.
Errors prevent further processing unless addressed.
However, users should take note of warnings as they may affect analyses downstream.

Metadata

An editable metadata template is extracted from the uploaded array files and can be downloaded by the user (**Download**) as a tab-delimited file (targets.txt) and edited in Excel to be re-uploaded by (**Upload Sample File**). Only tab-delimited file are accepted.

Each targets file requires a FileName column that corresponds to the array file names. It also requires a Name column, which should include unique values that will be used to identify each array downstream. Additional metadata columns, such as Disease state (most crucial), Age, Gender, and Race, can be added and modified at the user's discretion.

Any of the metadata columns can be selected to subset data into conditions (**Select Condition Column**). This is not an essential step; however, if applied, it is used to group samples in the resulting pre-processing plots, enhancing data visualisation. It is also helpful for assessing the reproducibility of replicates.
Click **Next >>** to view the next tab.

Probes

An editable Probes template is produced based on the uploaded array files. A default probe identification column will be assigned but can be altered using the **Probe Column** dropdown menu, the contents of which are linked to the annotation column in the Side Panel.
The probe template's 'Category' column is used to group the probes and annotate resulting pre-processing plots. This column can be edited by the user using the: **Control Probes** dropdown menu.
This enables users to identify control probes downstream; these will be removed after the normalisation step and are therefore not present in the final data outputs.
Probes to Remove dropdown menu enables the user to select

probes, such as EMPTY probes, that should be removed prior to normalisation.

Alternatively, the probe template can be downloaded (**Download**) as a tab-delimited file (probes.txt) and edited in Excel to be re-uploaded by the user (**Upload Probe File**). Additional annotations can be added to the Category column at the user's discretion to be labelled in resulting plots and or selected for removal.

Data Tables
The expression intensity and annotation data from uploaded array files are read into R, using the limma function 'read.maimages'. This creates an EListRaw object within which the foreground spots are weighted according to the spot filtering threshold described in the Array Properties section. Raw data can be inspected and visualised in this Data Tables tab via three subtabs:

- Within the **Foreground** and **Background** subtabs, users can visualise:
    - A **Table** which contains raw intensity expression values based on Array properties selected in the Side Panel,
    - **CV** data including tables, boxplots and density plots of replicate measurements for each probe,
    - A **Clustering** of the raw intensity values as a Heatmap or dendrogram

- Within the **Spot Filtering** subtab, users can visualise:
    - A weights **Table** which contains probe weights. Filtered spots are weighted as zero, while "good" spots are weighted as 1.
    - A **Heatmap** of the weighted spots. However, if no filtering is needed for the arrays a message is shown to show this.

Pre-Processing

This includes the pre-processing analysis of the raw data as described in Mowoe et al., (2021). Here data is spot filtered, background corrected, normalised, array weighted, and consolidated into a downloadable tab-delimited Datatable and an ExpressionSet which can be directly opened in R for further analysis outside of the app if desired. Data can be inspected and visualised in five subtabs:

- Within the **Raw Data** subtabs, users can visualise:
    - An **Expression Intensity Boxplot** of raw expression intensities. Data is grouped based on **Conditions** selected previously in the **Metadata** tab
    - **Clustering** showing hierarchical clustering of raw data as a heatmap or dendrogram.
- Within the **Background corrected** subtabs.
    - An **Expression Intensity Boxplot** of background corrected expression intensities.
    - A barplot of **Missing Values** that result from negative values produced by some background correction methods.

- **Clustering** showing hierarchical clustering of background corrected data as a heatmap or dendrogram.

- Within the **Normalised** subtabs.
    - An **Expression Intensity Boxplot** normalised expression intensities.
    - A boxplot and density plot of replicate probe **CV's** for each sample.
    - **Clustering** showing hierarchical clustering of normalised data as a heatmap or dendrogram.

Thus, users are able to visualise changes to the dataset after each pre-processing step.

- Within the **Array weight** subtab, users can visualise:
    - Calculated array weights in table form and as a barplot with data grouped based on **Conditions** selected.
- Within the **Final data** subtab, data has been pre-processed, previously selected control probes are removed, and data is consolidated using the means of replicate probes; users are able to visualise this as:
    - A **Table**, which contains pre-processed and consolidated expression intensity data
    - **Expression Intensity Boxplot** of pre-processed and consolidated expression intensities grouped based on the previously selected **Conditions**
    - A boxplot and density plot showing the **CV's** of the proteins across samples based on previously selected **Conditions**
    - A barplot which contains the percentage of **Missing values** in each array
    - An **MA plot** of the first array versus all the other arrays to give the user an idea of fold change within the dataset
    - Euclidean hierarchical **Clustering** of the data based on the proteins and the arrays to determine relationships.

Users may download data to be analysed elsewhere or move to the next tab for differential expression analysis within the app.

Differential Expression Analysis

Here, A linear model, using calculated array weights, fits the normalised data to fully model the systematic part of the data and determine variability between the groups. Contrasts matrices are then pulled from the metadata to determine the variability in the data based on comparisons of interest.

- The **Controls** dropdown allows users to select the condition to which all the other conditions should be compared. If no condition is selected here, all conditions will be compared to each other. Subsequently, an empirical Bayes method is used to moderate the standard errors of the estimated log-fold changes.
- The **p-value** dropdown allows the user to manually select or fill in a probability value below which probes will be considered

differentially expressed between conditions. Commonly used p-values include 0.001, 0.01, and 0.5
- The **Multiple Testing Correction** dropdown allows the user to select the procedure used to adjust the p-values for multiple testing.
- The **Fold change** dropdown allows the user to manually select or fill in the fold change at and above which probes will be considered differentially expressed between conditions.

In this tab users are able to visualise the data in two subtabs:

- The **Table** contains a dataframe with a row for the proteins and several colunms including
    - log fold change (logFC)
    - average expression (AveExpr)
    - moderated t statistic (t),
    - p-value,
    - adjusted p-value
    - log odds that the probe is differentially expressed (B), and a
    - threshold based on the p-value and fold change selected (TRUE: the proteins in significantly differentially expressed between conditions.)
- A **Volcano Plot**, which shows probes with large fold changes that may be of biological significance
- An interactive **MA plot** which potrays differential expression
- **Clustering**, which shows relationships between arrays and probes based on a euclidean hierarchical clustering

All tables are downloadable as tab-delimited datasets or ExpressionSets for use directly in R, and all plots are downloadable as vectorised png files

For further assistance contact

- Dr. S. Garnett (shaun.garnett@uct.ac.za)
- M. Mowoe (m_mowoe@yahoo.co.uk)
